# 3D Frequency-Domain Full Waveform Inversion for Whole-Breast Imaging With a Multi-Row Ring Array

**DOI:** 10.1109/ojuffc.2025.3570253

**Published:** 2025-05-14

**Authors:** REHMAN ALI, GAOFEI JIN, MELANIE SINGH, TREVOR MITCHAM, NEBOJSA DURIC

**Affiliations:** 1 Department of Imaging Sciences, University of Rochester Medical Center, Rochester, NY 14642 USA; 2 Department of Biomedical Engineering, University of Rochester, Rochester, NY 14627 USA

**Keywords:** Ultrasound tomography, full waveform inversion, 3D imaging, cylindrical waves

## Abstract

For ring-array ultrasound tomography, two-dimensional frequency-domain full waveform inversion is the clinical gold standard for high-resolution imaging of the breast. While yielding high-resolution images in the plane of the ring-array, the resulting slice-wise approach yields lower resolution out of plane when used to reconstruct the full volume. Instead, this work proposes a fully three-dimensional full-waveform inversion based on a multi-row ring-array transducer to improve out-of-plane resolution, while using cylindrical-wave transmissions to minimize acquisition and reconstruction time. For each numerical breast phantom tested, the root-mean-square error of three-dimensional full-waveform inversion is less than that of two-dimensional slice-wise full-waveform inversion by 6.3–13.7 m/s.

## I. INTRODUCTION

Ultrasound tomography (UST) uses the transmission of ultrasound through tissue to reconstruct high-resolution images of tissue properties such as sound speed and attenuation. The most common clinical use of UST is breast imaging [1] where sound speed and attenuation serve as biomarkers for cancer detection. Ring-array UST currently employs a two-dimensional (2D) full waveform inversion (FWI) reconstruction algorithm [2]. Although ultrasound physics is three dimensional (3D), elevational focusing enables current 2D modeling by confining the ultrasound beam to a thin volume.

However, as the frequency increases, real 3D physics deviates from the 2D model. Out-of-plane tissue variation can cause significant deviations from the 2D model that slicewise FWI cannot account for (e.g., the strong refraction at the sloping edge of the breast). To correct for this, recent work [3], [4] uses 3D FWI with an elevational focusing model to accurately account for the full diffraction in the tissue; despite applying 3D FWI, its achievable spatial resolution is limited by the lack of elevational sampling with a single row of receive elements. Although 3D FWI eliminates many artifacts caused by 2D modeling errors, partial volume effects and the lack of receive sampling outside the imaging plane limit the achievable spatial resolution of UST.

With the advent of cone-beam computed tomography (CBCT), X-ray computed tomography (CT) went from a 2D slicewise to a fully 3D imaging modality. We envision a similar trajectory for UST by expanding our current ring-array design [1] to include multiple rows of elements, thereby capturing the full 3D insonification of the tissue.

The main barrier to achieving a practicable 3D FWI with a multi-row ring-array transducer is computational cost. For example, time-domain FWI [3] would involve the complete 3D wavefield vs. time for each transmit event simulated. Frequency domain FWI significantly reduces the memory footprint by eliminating the time axis, making it much more practicable for 3D imaging. Although the paraxial approximation has been used to achieve fast 3D frequency-domain imaging based on plane-wave transmissions between a pair of opposing pitch-catch planar-array transducers [5], it cannot easily be applied to a ring-array geometry. Instead, this work relies on the convergent Born-series solution to the full 3D Helmholtz equation [6] to perform 3D frequency-domain FWI without any approximations.

Although the pitch-catch setup [5] also achieves 3D imaging, its motorized rotation may lengthen acquisition times and introduce motion artifacts. A multi-row ring-array would reduce acquisition time by eliminating the need for rotation. Additionally, cylindrical wave transmits (as opposed to single-element transmits) keep the number of transmissions to a minimum, thereby reducing acquisition and reconstruction times even further. By using this transmission scheme with multiple rows of receivers, elevational focusing is no longer necessary. Rather than confine the ultrasound beam to a thin slice, our approach uses broad unfocused cylindrical-wave transmits to efficiently sample the full volume. This work provides an initial proof-of-concept demonstration with numerical breast phantoms to motivate the development of a multi-row ring-array transducer for 3D UST imaging.

## II. THEORY

### A. FREQUENCY-DOMAIN FWI USING THE HELMHOLTZ EQUATION

We solve the 3D Helmholtz equation to obtain a pressure field u for a given source δ, frequency ω=2πf, and slowness s (i.e., the reciprocal of sound speed):

(1) $$\left(\nabla^{2}+\omega^{2}s^{2}\right)u\left(\omega ,s\right)=\delta \left(\omega \right),$$

If we vectorize the pressure field and source over spatial locations on a discretized grid as u and δ, respectively, we abstractly denote the ($\nabla^{2}+\omega^{2}s^{2}$) operator as a matrix A applied to u, which equals to δ:

(2) $$A\left(\omega ,s\right)u\left(\omega ,s\right)=\delta \left(\omega \right),$$

Differentiating both sides with respect to s yields:

(3) $$\frac{\partial u}{\partial s}=-A^{-1}\frac{\partial A}{\partial s}u=-A^{-1}\frac{\partial A}{\partial s}A^{-1}\delta ,$$

Rather than observing the full pressure field u at all locations in space, we only record the pressure field p at the ring array. The relationship between p and u is: p=Ku, where K is a sampling operator that extracts the pressure field at each element of the ring array, treating elements as point receivers. Modeling the elements as point sources and receivers has previously been validated and applied to *in vivo* data [2]. The relationship between changes in slowness and changes in the simulated pressure field at the ring array is

(4) $$\frac{\partial p}{\partial s}=-KA^{-1}\frac{\partial A}{\partial s}u=-KA^{-1}\frac{\partial A}{\partial s}A^{-1}\delta ,$$

Frequency-domain FWI minimizes the following cost function with respect to the slowness image s:

(5) $$E\left(\omega ,s\right)=\frac{1}{2}\sum_{i=1}^{N}{\left\|p_{i}\left(\omega ,s\right)-p_{obs,i}\left(\omega \right)\right\|}_{2}^{2},$$

where $p_{i}$ and $p_{\text{obs,i}}$ are the simulated and observed pressure measurements on the ring array for the $i^{\text{th}}$ cylindrical wave transmission $\delta_{i}$ (i ranges from 1 to N, the number of cylindrical waves). The gradient of E(ω,s) with respect to slowness s is given by:

(6) $$\nabla_{s}E\;=\sum_{i=1}^{N}Re\left\{{\left(\frac{\partial p_{i}}{\partial s}\right)}^{H}\left(p_{i}-p_{obs,i}\right)\right\}\;=-\sum_{i=1}^{N}Re\left\{{\left(\frac{\partial A}{\partial s}u_{i}\right)}^{H}{\left(A^{H}\right)}^{-1}K^{T}\left(p_{i}-p_{obs,i}\right)\right\}\;=-\sum_{i=1}^{N}Re\left\{{\left(2\omega^{2}s⊙u_{i}\right)}^{H}{\left(A^{H}\right)}^{-1}K^{T}\left(p_{i}-p_{obs,i}\right)\right\},$$

where ⊙ refers to the point-wise multiplication between the vectors. This definition of the gradient is used to implement the conjugate gradient method as done in prior work [2]. Solutions $A^{-1}$ and ${\left(A^{H}\right)}^{-1}$ to the Helmholtz equation and its adjoint, respectively, are achieved via the convergent Born series solution to the Helmholtz equation [6]. The theory behind frequency-domain FWI [2] does not change in any significant way when moving from 2D to 3D.

### B. COMPLEX SOURCE ESTIMATION

The amplitude and phase of each source is not known *a priori* and is estimated at each iteration of FWI. The following projection formula provides a complex scaling $\gamma_{i}$ that captures both the amplitude and phase of each source:

(7) $$\gamma_{i}=\frac{{p_{i}}^{H}p_{obs,i}}{{p_{i}}^{H}p_{i}}$$

The source vector and pressure fields are scaled to $\left\{\delta_{i}\to \right.\left.\gamma_{i}\delta_{i}\right\},\left\{u_{i}\to \gamma_{i}u_{i}\right\}$, and $\left\{p_{i}\to \gamma_{i}p_{i}\right\}$, respectively. The same source estimation and scaling process is in used in [2].

## III. METHODS

Receive channel data from cylindrical-wave transmits using a multi-row ring-array transducer (22-cm diameter; 32 rows of 256 circumferential elements; 2.4 mm spacing between rows) was simulated in k-Wave [7] for three different 3D numerical breast phantoms. A total of 256 cylindrical wave transmits (0.75 MHz center frequency; 67% fractional bandwidth) were produced by simultaneously firing the same circumferential element in each row. Signals were received by the full 256 × 32 array of elements, resulting in 8192 recorded signals per cylindrical wave transmission. The resulting channel data (with −20 dB Gaussian white noise added) was used to reconstruct the full 3D profile of sound speed in the breast. The first breast phantom was developed in [4] from the Virtual Imaging Clinical Trial for Regulatory Evaluation (VICTRE) repository, and the next two breast phantoms were adapted from dynamic contrast-enhanced MRI images [8]

We compared 3D FWI to 2D slicewise FWI based on receive channel data from each row (or slice) of the ring. By assuming that there is no elevational variation in sound speed, 2D FWI could be applied to each row of received signals (256 recorded signals per cylindrical wave transmit per row) by treating each row of receivers as sampling a single slice of the full volume. Each FWI was performed from 200 to 800 kHz in 50 kHz steps with 5 iterations of conjugate gradient at each frequency. A homogeneous 1500 m/s starting model was used in each case. The improvement in image reconstruction from 2D slicewise to 3D FWI was evaluated using root-mean-square error (RMSE) and Pearson,s correlation coefficient (PCC) with the ground truth.

## IV. RESULTS

**FIGURE 1.**
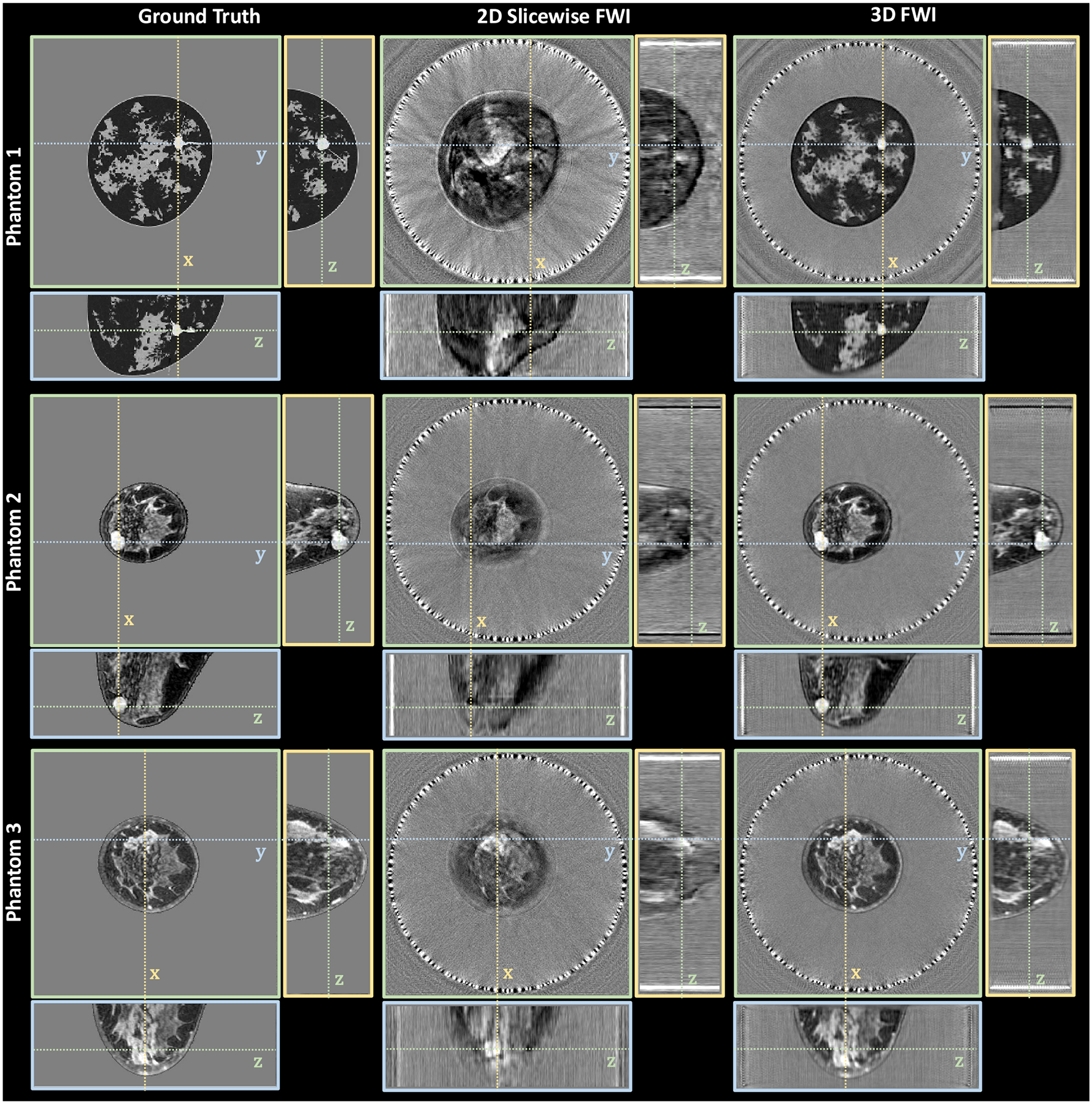
Comparison of 2D slicewise to 3D FWI with simulated multi-row ring array. Cylindrical wave transmits from a multi-row ring-array (32 rows; 256 elements per row; 22 cm diameter; 2.4 mm between rows) were simulated in three different numerical breast phantoms. Orthographic slice views of the reconstructed volumes intersect at suspicious high sound speed masses in each phantom (cancers in phantoms 1 and 2, and dense breast tissue in phantom 3). Each sound speed images is displayed in grayscale from 1400 to 1600 m/s: (Left Column) ground-truth sound speed image; (Middle Column) volume reconstructed using 2D slicewise FWI; (Right Column) volume reconstructed using 3D FWI.

Figure 1 demonstrates several improvements in the reconstructed image visible across different imaging planes in each of the three numerical breast phantoms when moving from 2D slicewise to fully 3D FWI. Table 1 report the RMSE in sound speed reconstruction and the PCC with the ground truth. Across all the phantoms, the RMSE of 3D FWI is less than that of 2D slicewise FWI by 6.3–9.2 m/s. Similarly, the PCC of 3D FWI is greater than that of 2D slicewise FWI by 0.2065–0.3690. Most notably, in both phantoms 1 and 2, 2D slicewise FWI completely misses the cancerous lesions that is well visualized by 3D FWI across all three imaging planes. Additionally, the coronal view produced by 2D FWI in phantom 3 shows a globular region of high sound speed that could be mistaken for a cancerous mass. However, the 3D FWI better reconstructs the shape of this region of high sound speed, revealing it to be dense breast tissue.

In practice, 2D slicewise imaging is more accurate than shown here because the single-row ring array currently used in UST is elevation-focused into the imaging plane with a much smaller elevation height. Therefore, we expect 2D slicewise FWI with an elevation-focused ring-array transducer [3] to be less susceptible to artifacts caused by out-of-plane sound speed variation than with cylindrical wave transmits from a multi-row ring array. Nevertheless, cylindrical wave transmits sampled by a 256 × 32 receiving array makes better use of 3D FWI by reconstructing at higher spatial resolution than the single-row ring-array.

## V. DISCUSSION

This simulation study demonstrates the feasibility of 3D breast imaging with a multi-row ring-array transducer. This raises questions about manufacturing such a high-channel count system. Butterfly iQ is known to use a 2D array of 9000 capacitive micromachined ultrasound transducers (CMUTs) [9]. Whereas piezoelectric crystals require significant external circuitry for such large arrays, CMUTs integrate electronics directly on-chip, enabling greater scalability. Therefore, it may be possible to use CMUT technology to achieve such high channel counts. Furthermore, Photo-Sound’s LEGION Hub [10] allows 16 different 256-channel systems to be operated in parallel, leading to a total of 4096 channels. With 2:1 multiplexing, a complete 256 × 32 ring array (8192 elements) could be accessed. Alternatively, the ring array may be limited to 16 rows (4096 elements) and translated vertically to scan the full 3D volume.

Another consideration is computational cost. Multi-GPU systems can reduce computation time by parallelizing 3D FWI. Dedicated integrated circuits could apply fast Fourier transforms to raw data and output specific frequencies used in FWI to reduce dataset size. By establishing the feasibility of 3D FWI with a multi-row ring-array, we aim to improve computational efficiency in future work and motivate the development of a multi-row ring array transducer.

## VI. CONCLUSION

This preliminary *in-silico* demonstration of 3D FWI with cylindrical wave transmits serves to foreshadow the next generation of ring-array breast UST system. By adopting a multi-row ring geometry, we can overcome the limitations of 2D slicewise FWI associated with current ring-array UST and avoid the drawbacks of mechanical rotation associated with the current implementation of 3D UST. Ultimately, the goal of this work is to motivate the development of a physical multi-row ring-array transducer and acquisition system for 3D FWI imaging of the breast. The code used to simulate phantoms 2 and 3 and reconstruct their corresponding 3D volumes (based on both 2D slicewise and 3D FWI) have been made publicly available at https://github.com/rehmanali1994/3D-FWI-MultiRowRingArrayUST (DOI: https://zenodo.org/badge/latestdoi/871745761).

## Figures and Tables

**TABLE 1.** RMSE and PCC of the reconstructed sound speed volume with the ground truth for each numerical breast phantom in figure 1.

	2D Slicewise FWI		3D FWI	
	RMSE	PCC	RMSE	PCC
Phantom 1	29.2 m/s	0.6220	15.5 m/s	0.8848
Phantom 2	17.6 m/s	0.6921	10.1 m/s	0.8981
Phantom 3	14.7 m/s	0.6857	8.4 m/s	0.8967

## References

[1] Sandhu GY, Li C, Roy O, Schmidt S, and Duric N, “Frequency domain ultrasound waveform tomography: Breast imaging using a ring transducer,” Phys. Med. Biol, vol. 60, no. 14, pp. 5381–5398, Jul. 2015. 26110909 10.1088/0031-9155/60/14/5381 PMC4902020

[2] Ali R , “2-D slicewise waveform inversion of sound speed and acoustic attenuation for ring array ultrasound tomography based on a block LU solver,” IEEE Trans. Med. Imag, vol. 43, no. 8, pp. 2988–3000, Aug. 2024. 10.1109/TMI.2024.3383816 PMC11294001 38564345

[3] Li F, Villa U, Duric N, and Anastasio MA, “A forward model incorporating elevation-focused transducer properties for 3D full-waveform inversion in ultrasound computed tomography,” IEEE Trans. Ultrason., Ferroelectr., Freq. control, vol. 70, no. 10, pp. 1339–1354, Oct. 2023. 37682648 10.1109/TUFFC.2023.3313549 PMC10775680

[4] Li F, Villa U, Park S, and Anastasio MA, “3-D stochastic numerical breast phantoms for enabling virtual imaging trials of ultrasound computed tomography,” IEEE Trans. Ultrason., Ferroelectr., Freq. Control, vol. 69, no. 1, pp. 135–146, Jan. 2022. 34520354 10.1109/TUFFC.2021.3112544 PMC8790767

[5] Wiskin JW, Borup DT, Iuanow E, Klock J, and Lenox MW, “3-D nonlinear acoustic inverse scattering: Algorithm and quantitative results,” IEEE Trans. Ultrason., Ferroelectr., Freq. Control, vol. 64, no. 8, pp. 1161–1174, Aug. 2017. 28541199 10.1109/TUFFC.2017.2706189 PMC6214813

[6] Osnabrugge G, Leedumrongwatthanakun S, and Vellekoop IM, “A convergent born series for solving the inhomogeneous Helmholtz equation in arbitrarily large media,” J. Comput. Phys, vol. 322, pp. 113–124, Oct. 2016.

[7] Treeby BE and Cox BT, “K-wave: MATLAB toolbox for the simulation and reconstruction of photoacoustic wave fields,” J. Biomed. Opt, vol. 15, no. 2, 2010, Art. no. 021314. 10.1117/1.3360308 20459236

[8] Saha A , “A machine learning approach to radiogenomics of breast cancer: A study of 922 subjects and 529 DCE-MRI features,”Brit. J. Cancer, vol. 119, no. 4, pp. 508–516, 2018. 30033447 10.1038/s41416-018-0185-8 PMC6134102

[9] Liu JY, Xu J, Forsberg F, and Liu J-B, “CMUT/CMOS-based butterfly iQ—A portable personal sonoscope,” Adv. Ultrasound Diagnosis Therapy, vol. 3, no. 3, pp. 115–118, 2019.

[10] Thompson W, Brecht H-P, Ermilov SA, and Ivanov V, “Massive parallel ultrasound and photoacoustic PC-based system,” Proc. SPIE, vol. 11240, p. 159, Feb. 2020.

